# Comparative Study of the Efficacy of EHO-85, a Hydrogel Containing Olive Tree (*Olea europaea*) Leaf Extract, in Skin Wound Healing

**DOI:** 10.3390/ijms241713328

**Published:** 2023-08-28

**Authors:** Bárbara Torrecillas-Baena, Marta Camacho-Cardenosa, María Dolores Carmona-Luque, Gabriel Dorado, Miriam Berenguer-Pérez, José Manuel Quesada-Gómez, María Ángeles Gálvez-Moreno, Antonio Casado-Díaz

**Affiliations:** 1 Maimonides Institute of Biomedical Research in Cordoba (IMIBIC), 14004 Cordoba, Spain; b42tobab@uco.es (B.T.-B.); marta.camacho@imibic.org (M.C.-C.); mariadolores.carmona@imibic.org (M.D.C.-L.); md1qugoj@uco.es (J.M.Q.-G.); 2 Endocrinology and Nutrition Unit, Reina Sofia University Hospital, 14004 Cordoba, Spain; 3 CIBER Fragilidad y Envejecimiento Saludable (CIBERFES), Instituto de Salud Carlos III, 28029 Madrid, Spain; bb1dopeg@uco.es; 4 Cellular Therapy Unit, Reina Sofia University Hospital, 14004 Cordoba, Spain; 5 Department Bioquímica y Biología Molecular, Campus Rabanales C6-1-E17, Campus de Excelencia Internacional Agroalimentario (ceiA3), Universidad de Córdoba, 14071 Córdoba, Spain; 6 Department of Community Nursing, Preventive Medicine and Public Health and History of Science, University of Alicante, San Vicente del Raspeig, 03690 Alicante, Spain; miriam.berenguer@ua.es

**Keywords:** hydrogel, *Olea europaea* leaf extract, oleuropein, EHO-85, dexpanthenol, *Centella asiatica*, hyaluronic acid, wound healing, skin

## Abstract

Olive tree (*Olea europaea*) leaf extract (OELE) has important antioxidant and anti-inflammatory properties, supporting its use in human clinical practice. We recently designed an amorphous hydrogel called EHO-85 (EHO indicates olive leaf extract in Spanish) containing OELE for skin ulcer treatments. Yet, its effectiveness has not been previously compared with other products used in routine clinical practice. This is necessary to evaluate its potential translation to the human clinic. Thus, in this study, the effect of EHO-85 on healing was evaluated in comparison with treatments containing Indian/Asiatic pennywort (*Centella asiatica*), hyaluronic acid, or dexpanthenol in a rat model. The speed of wound closure and histological parameters after seven and 14 days were analyzed. All treatments accelerated wound closure, but there were differences between them. Dexpanthenol after seven days produced the highest epithelialization and the lowest inflammation and vascularization. EHO-85 also promoted epithelialization and reduced vascularization. After 14 days, wounds treated with EHO-85 showed less inflammation and higher levels of collagen in the extracellular matrix. This indicates a higher degree of maturity in the regenerated tissue. In conclusion, the effect of EHO-85 on healing was equal to or superior to that of other treatments routinely used in human clinical practice. Therefore, these results, together with previous data on the effects of this hydrogel on ulcer healing in humans, indicate that EHO-85 is a suitable, low-cost, and efficient therapeutic option for wound healing.

## 1. Introduction

The skin is the largest organ of the body, having various and diverse functions, including being a multifunctional physical barrier, protecting against mechanical, thermal, and other physical injuries, as well as hazardous substances. Additionally, it possesses sensory and immune properties. In summary, it protects the body from external damage, including environmental changes, while maintaining physiological homeostasis [1].

Cutaneous wound healing mechanisms are activated immediately after the occurrence of an injury, which disrupts the continuity of the surface [2]. Normally, in not-too-severe abrasions, trauma, acute surgical wounds, etc., heals are efficiently repaired [3]. However, aging, infections or certain underlying medical conditions, such as diabetes or obesity, may interfere with wound healing processes, conditioning wound chronification [4,5].

Due to population aging and the increasing incidence of diabetes and obesity worldwide, the problems resulting from acute or chronic wounds have dramatically increased. Indeed, they affect more than 305 million people in the world. This generates, in addition to the physical and mental suffering of those who suffer from them, a financial burden of management that will reach $80 milliards by 2024 [6].

With the aim of improving the natural mechanisms of the wound repair process, numerous therapies have been developed, including pharmacological ones [7]. The most important disadvantages of many pharmacological agents are their systemic use and relatively high dose requirements, which means that they may have risks and other potential adverse or side effects, thus reducing their efficacy in healing [8].

To circumvent these restrictions, multiple types of efficient, cost-effective, and minimally-invasive dressings are widely employed, to treat skin wounds [9]. These dressings have the advantage that they are easy to use, preserve hydration within the wound, protect against infection, prevent disruption of the wound base, and are painless to remove. In addition, different types of biomaterials can be used in their composition to facilitate the delivery and activation of agents. That allows manipulation of the biological microenvironment of the wound by actively promoting mechanisms that stimulate tissue regeneration [10].

Among the active compounds that are gaining importance in promoting wound healing are a wide variety of natural products, like plant extracts. These are characterized by containing numerous bioactive compounds with antimicrobial, immunoregulatory and antioxidant properties [11,12,13]. Thus, interesting therapeutic properties have been extensively reported for OELE, partly related to its antioxidant and anti-inflammatory capacities [14,15]. These properties are mainly due to its high polyphenolic content, with oleuropein being the most abundant one, followed by hydroxytyrosol and tyrosol [16]. Moreover, the antioxidant activity of these compounds is synergistic [17]. Indeed, the application of oleuropein, or OELE, to wounds significantly accelerated the healing of skin ulcers, largely mediated by its antioxidant and anti-inflammatory properties [18,19,20].

Different dressings have been designed with suitable properties to create microenvironments that promote healing while carrying biologically active agents that accelerate healing. Several strategies have been used to optimize their water stability/mechanical properties, as well as their biological performance. Among those containing hyaluronic acid, extracts from Indian pennywort/Asiatic pennywort *(Centella asiatica*) and dexpanthenol stand out for their excellent results [21,22,23,24].

Many of these dressings come in the form of creams or ointments. However, several studies have pointed out that the use of hydrogels can create more favorable conditions for healing because they are less occlusive and do not contain hydrophobic compounds, such as oils, in their composition. Thus, they support better gas exchange and moisture supply [25,26]. Hydrogels, due to their versatility in composition and ability to encapsulate different agents such as drugs, growth factors, nanovesicles, and cells, among others, have high clinical potential. Therefore, in the last decade, the study and applications of these biomaterials in different clinical fields have exponentially grown. These include drug delivery, cell therapy, wound healing, and tissue regeneration, among others [27]. Recently, it has been reported that EHO-85, an amorphous hydrogel containing OELE, modulates the wound microenvironment through its antioxidant effect as well as regulation of pH and wetting [14]. That way, it holistically cooperates with natural physiological processes involved in wound healing [28]. With these properties, EHO-85 has been found to have superior wound healing rates than standard amorphous hydrogel treatments, also accelerating the healing of difficult-to-heal wounds [29].

Therefore, on this background, the aim of the present study is to evaluate the healing capacity of the amorphous hydrogel EHO-85 in comparison with standard topical treatments commonly used in clinical practice in a rat model of excisional wounds. For this purpose, we have used: (i) a negative control (without topical treatment); and (ii) three topical treatments as positive controls, whose active ingredients are *Centella asiatica* extract, hyaluronic acid, and dexpanthenol.

## 2. Results

### 2.1. Temporal Evaluation of Wound Closure

**Figure 1:**
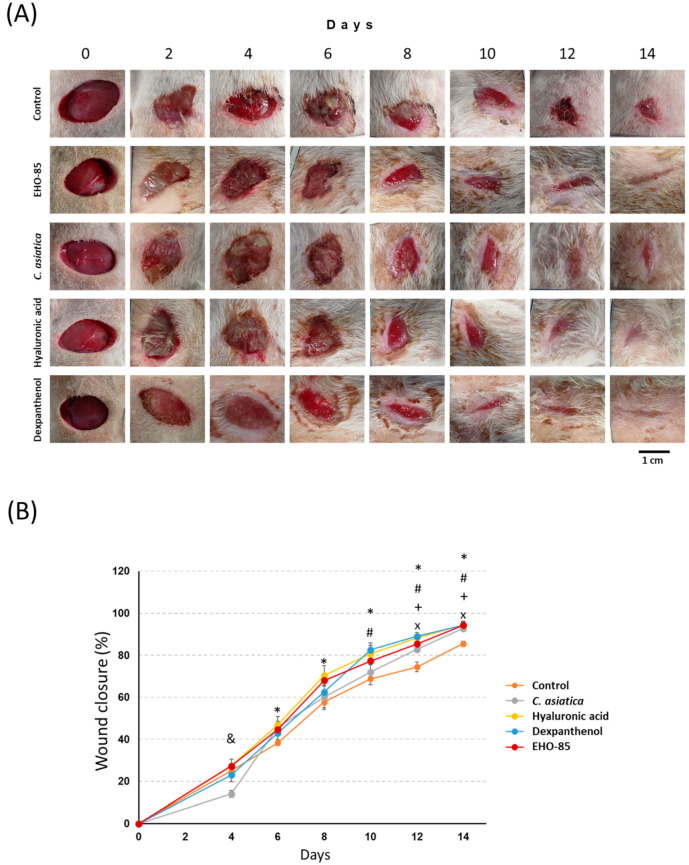
Effects of different treatments on wound closure over time. (**A**), representative images of the temporal evolution of wound sizes subjected to different treatments. (**B**) Quantification of percentages of wound closure in different treatments. *: *p* < 0.05 for hyaluronic acid vs. control; #: *p* < 0.05 for dexpanthenol vs. control; +: *p* < 0.05 for EHO-85 vs. control; x: *p* < 0.05 for *C. asiatica* vs. control; and &: *p* < 0.05 for *C. asiatica* vs. the rest of the treatments.

Figure 1A shows representative images of wound-closure evolution after different treatments. At the end of the study (14 days), it was observed that all treatments caused faster wound closure than the control. The wounds with the most advanced state of healing were those treated with dexpanthenol, hyaluronic acid, and EHO-85.

Quantification of wound closure over time is shown in Figure 1B. After six to eight days, the different treatments used produced a greater degree of wound closure as compared to the untreated control. These differences were statistically significant (*p* < 0.05) after six days in the case of hyaluronic acid, after 10 days for dexpanthenol, and after 12 and 14 days with EHO-85 amorphous hydrogel and *Centella asiatica*, respectively. At the end of the study, treatments with EHO-85, hyaluronic acid, and dexpanthenol produced the highest percentages of wound closure. They were 94.3%, 94.4%, and 94.3% of the average, respectively, compared to 86% of the control group. Treatment with *C. asiatica* produced an average wound closure of 92.7%, slightly lower than that achieved with the other treatments, albeit not statistically significant. Among the four evaluated products, no significant differences were observed in any of the times except for *C. asiatica* after four days, showing lower wound closure than those other treatments (Figure 1B).

### 2.2. Histological Analyses after Seven Days of Treatments

**Figure 2:**
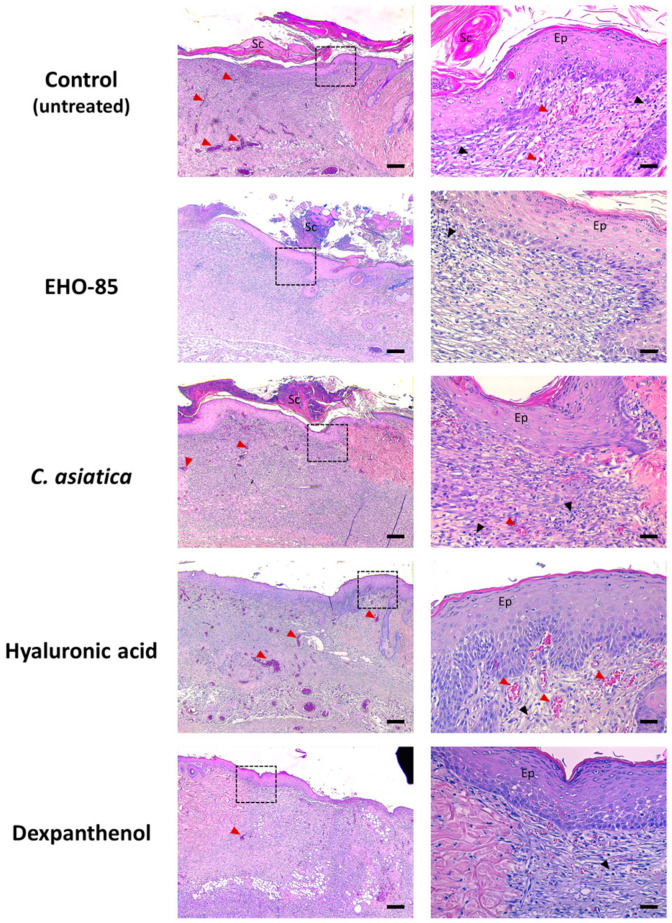
Representative images of histological sections of wounds stained with hematoxylin-eosin after seven days of treatment. The images on the left correspond to each treatment taken at 40× magnification (bars: 200 μm). Those on the right are images at 200× (bars: 40 μm) of details of the previous ones (dotted box in each image). Red and black arrows indicate blood vessels and areas with the presence of inflammatory cells, respectively. Sc: Scab; and Ep: Epidermis.

After seven days of treatments, wound tissue samples were histologically analyzed for various parameters after hematoxylin-eosin (H & E) staining (Figure 2). In general, scabs, thickened epidermis, and a moderate or abundant number of inflammatory cells were observed in all treatments. 

**Figure 3:**
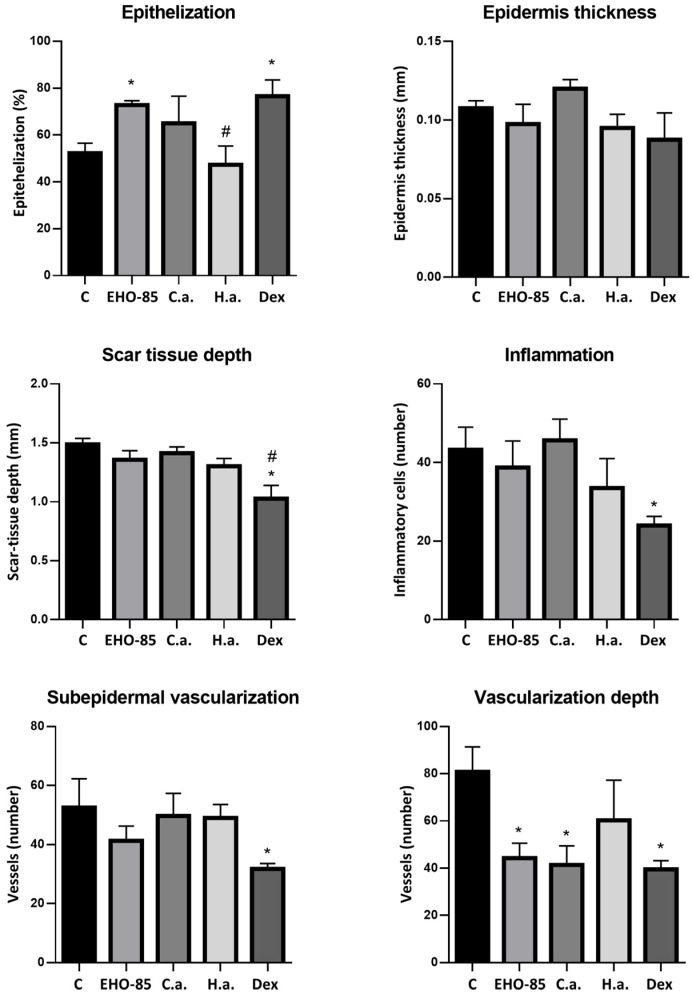
Quantification of histological parameters evaluated after seven days of treatments. C: control (untreated); C.a.: *Centella asiatica*; H.a.: Hyaluronic acid; and Dex: Dexpanthenol. *: *p* < 0.05 vs. control; and #: *p* < 0.05 vs. EHO-85.

Quantification of different evaluated parameters is shown in Figure 3. It can be seen that, with respect to control wounds, those treated with EHO-85 and dexpanthenol presented the highest degree of epithelialization (*p* < 0.05). With the latter treatment, a significant decrease in the depth of cicatricial tissue, inflammation, and vascularization were also observed, both at the subepidermal level and in depth. Regarding the latter parameter, a decrease was also observed in wounds treated with EHO-85 or *C. asiatica*. Hyaluronic acid did not significantly affect any evaluated parameter, although a tendency to decrease the number of infiltrating inflammatory cells was observed (Figure 3).

### 2.3. Histological Analyses after 14 Days of Treatments

After 14 days of treatments, the degree of wound closure was very high in all instances. Therefore, in addition to parameters related to epithelialization, inflammation, and vascularization, wound samples were histologically analyzed for collagen levels in the extracellular matrix as an indicator of the maturity of regenerated tissues.

**Figure 4:**
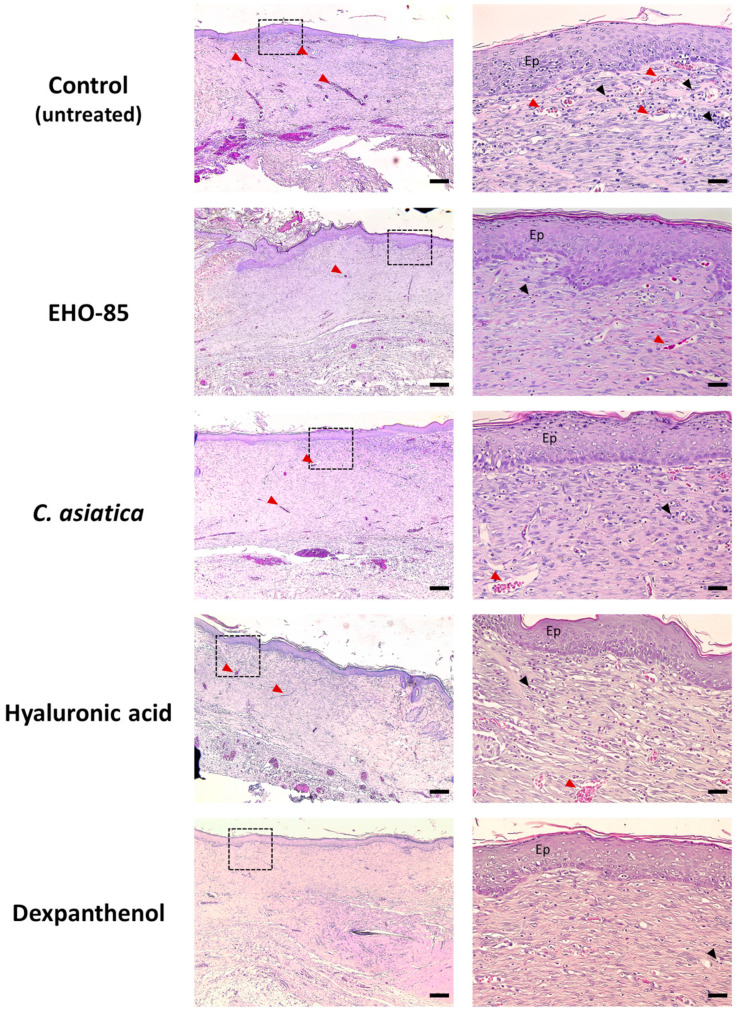
Representative images of histological sections of wounds stained with hematoxylin-eosin after 14 days of treatment. See the legend in Figure 2.

Figure 4 shows representative images of histological sections stained with H & E of wounds after 14 days of being subjected to different treatments. All treatments showed a high degree of epithelialization, with a thinner epidermis than after seven days. This was accompanied by a decrease in inflammatory cell infiltration and vascularization. All this was an indicator that the healing process was in a phase of maturation of the dermis.

**Figure 5:**
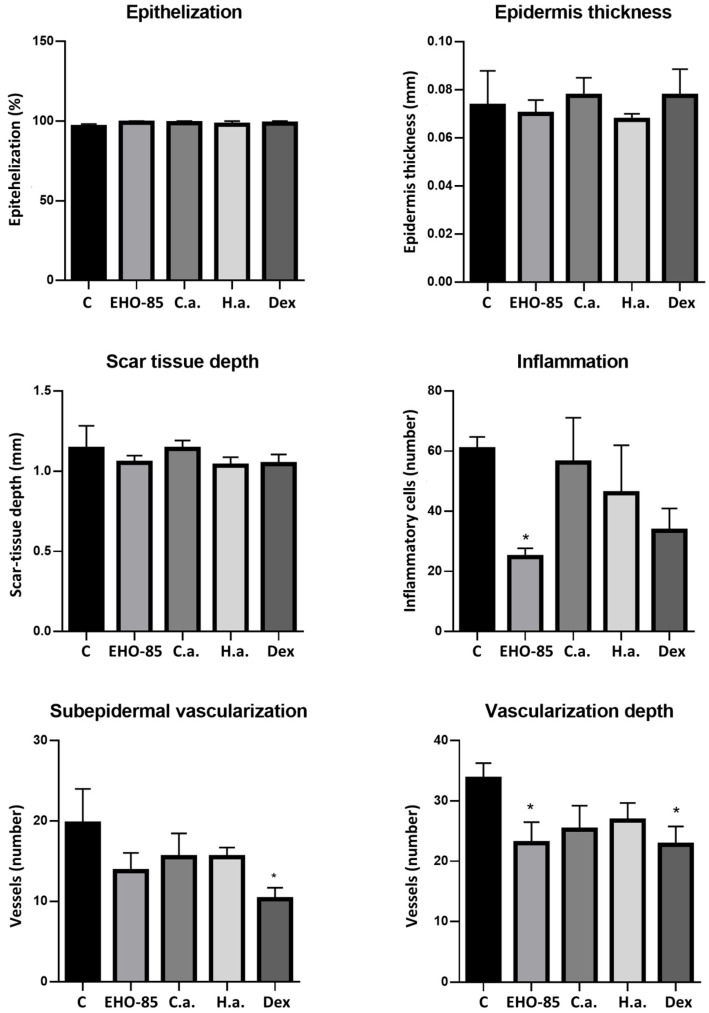
Quantification of histological parameters evaluated after 14 days of treatments. C: Control (untreated); C.a.: *Centella asiatica*; H.a.: Hyaluronic acid; and Dex: Dexpanthenol. *: *p* < 0.05 vs. control.

Quantification of parameters evaluated in the wounds showed that, for epithelialization, epidermal thickness, and scar tissue depth, no significant differences were observed between different treatments, including the control one. However, in relation to inflammatory cell infiltration, treatment with EHO-85 produced a reduction with respect to the control and the other treatments (Figure 5). All treatments reduced vascularization with respect to control. These decreases were significant in the case of treatment with dexpanthenol, in both the subepidermal and deeper regions. For the latter, treatment with EHO-85 also produced a significant decrease. The lower vascularization observed with treatments, mainly dexpanthenol and EHO-85, suggests a greater maturation of the healing tissue.

**Figure 6:**
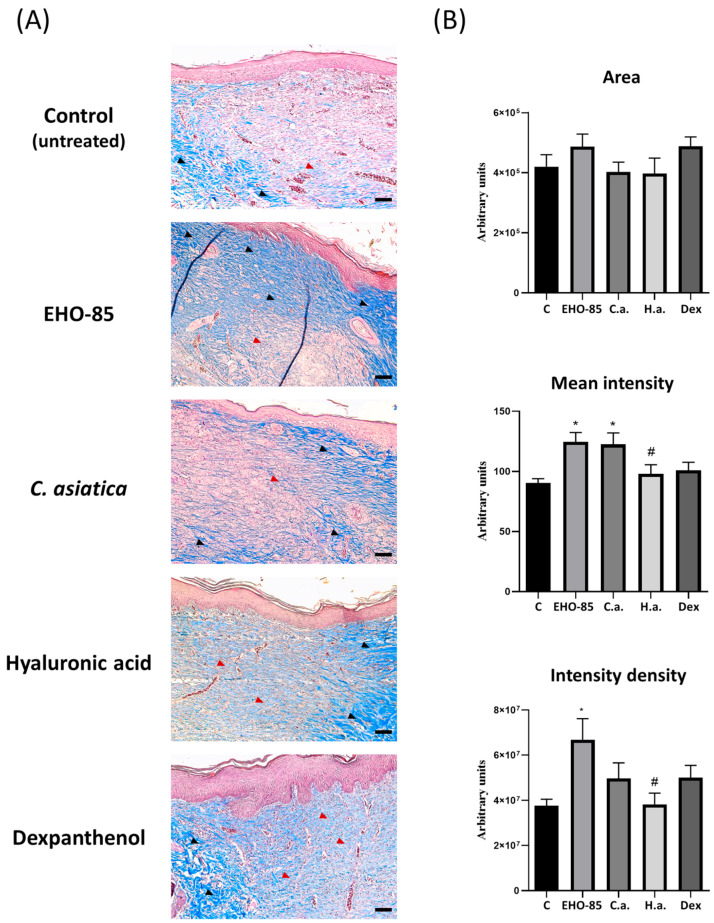
Masson’s trichrome staining of collagen in histological sections of wounds after 14 days of treatment. (**A**) Representative light microscope images at 100× (bars: 40 μm) of each treatment. The blue color indicates the presence of collagen. The intensity of the color is proportional to the collagen density. Black and red arrows indicate regions with high or low collagen density, respectively. (**B**) Quantification of staining area, mean intensity, and density of collagen staining, obtained through image analyses with ImageJ. C: control (untreated); C.a.: *Centella asiatica*; H.a.: Hyaluronic acid; and Dex: Dexpanthenol. *: *p* < 0.05 vs. control; and #: *p* < 0.05 vs. EHO-85.

In relation to this, collagen content was analyzed by Masson’s trichrome staining. Such a procedure generates a blue color, the intensity of which is proportional to the concentration of this protein in the extracellular matrix. Figure 6A shows representative images of histological sections using wound samples subjected to different treatments after 14 days. In all of them, unstained or light-blue-stained areas of the dermis were observed, indicating a lower deposition of collagen in the extracellular matrix. That indicates a lack of maturity in the regenerated tissue. The highest intensity of collagen fibers was observed in sections from wounds treated with EHO-85. Quantification of collagen staining by image analysis in relation to the area occupied by collagen showed that it was greater in treatments with EHO-85 and dexpanthenol. Nevertheless, differences were not statistically significant with respect to the other treatments. However, in terms of color intensity, both the mean intensity and cumulative density were higher in wounds treated with EHO-85. This indicates a higher collagen content and therefore a higher degree of maturity of the extracellular matrix than the other evaluated treatments (Figure 6B).

## 3. Discussion

In the present study, we have shown that EHO-85 amorphous hydrogel has a capacity equal to or superior to other well-established products on the market to promote the healing of cutaneous wounds. Other products tested were extracts of *Centella asiatica*, hyaluronic acid, or dexpanthenol, either in creams or ointments. Therefore, their structure is different from that of EHO-85 hydrogel. Hydrogels consist of hydrophilic polymers and usually contain more than 90% water. In medicine, they are being used in different applications, such as wound treatment, cell therapy, and drug delivery, among others [27]. The properties of hydrogels make them ideal for wound treatment because they maintain moisture, do not stick, are gas-permeable, and are biocompatible, favoring exudate absorption [25]. In previous studies, we have shown that EHO-85 satisfies these properties, in addition to possessing significant antioxidant activity, due to its OELE content [14,28]. This explains its positive effects on wound healing at the clinical level [29].

In addition, the composition of EHO-85 ensures adequate biocompatibility for use in skin wounds. Thus, carbopol 980, used as a polymer to create the hydrogel base, has been widely used in skin product formulations. Among them are hydrogels containing natural compounds [30]. On the other hand, triethanolamine is a common neutralizing agent used for polymer gelation. Additionally, Geogard Ultra (gluconolactone and sodium benzoate) and EDTA are commonly used as preservatives in dermocosmetics. They were included in the formulation to avoid microbiological contamination [31]. Glycerin and fucocert are two other compounds, widely used in skin care products, for their moisturizing capacity. The latter is proven to help create a protective film on the wound [32]. With regard to the OELE used in the composition of the hydrogel, studies by our group have shown in vitro that it has the capacity to protect skin cells, such as fibroblasts and keratinocytes, from oxidative stress induced by hydrogen peroxide [28]. In addition, several studies have shown that oleuropein, the main polyphenol in olive-tree leaf extract, promotes proliferation or has no cytotoxic effects in fibroblast cultures [33,34]. Therefore, the combined properties of the hydrogel components favor its biocompatibility in the treatment of skin ulcers.

On the other hand, creams are emulsions of water in oil (oily creams) or oil in water (vanishing creams), in which the active ingredient is dispersed between the oil and water phases. Thus, they have been defined as semi-solid emulsions containing more than 20% water and volatiles and/or less than 50% hydrocarbons, waxes, or polyethylene glycols as vehicles. The oil content is higher in ointments, and therefore creams are less fatty, viscous, and moisturizing but more spreadable than ointments. Creams have the advantage that they can incorporate hydrophilic or hydrophobic active ingredients, which can be released when topically applied to the skin [25]. However, for wound treatment, it is suggested that hydrogels have a greater capacity than creams to retain moisture, favor cell migration, and protect wounds [25]. Hydrogels can also be dried and transformed into sponges, which can be used as wound dressings. Thus, a biosponge derived from a hydrogel (based on carboxymethyl chitosan/poly-γ-glutamic acid/platelet-rich plasma) has recently been described. This sponge placed in full-thickness skin defects in mice had the capacity to release growth factors and promote healing [35]. Indeed, a recent study showed that out of five galenic preparations (three hydrogels, a semi-occlusive ointment, and petrolatum), the former presented greater hydration and tissue breathability in a test on pig-ear skin. Thus, the authors suggested that hydrogels are a better vehicle for the treatment of skin wounds [26].

These data support our results, in which we have found that certain parameters of wound healing, such as collagen formation and inflammation, progress more favorably when wounds are treated with EHO-85. However, other authors have also shown that an ointment containing 1% aqueous olive-tree leaf extract accelerated healing in a rat model, similar to an ointment containing 1% *Centella asiatica* extract [18]. However, in this study, unlike our results, the treatment with the reference compound (*C. asiatica*) produced a better performance at the level of epithelialization, wound closure, and the presence of mature hair follicles at the end of the experiment with respect to the product with OELE extract [18]. This suggests that the application of OELE in hydrogel form may be more effective for wound treatment.

Regarding wound closure, it was accelerated with all the products tested with respect to the untreated control. This is in agreement with the previous results of our group and what has been described by other authors in the case of hyaluronic acid, *Centella asiatica*, and dexpanthenol [14,36,37,38]. Among the four products tested, no significant differences in the speed of wound closure were detected. However, while the differences with respect to the control were significant after six days of treatment with hyaluronic acid, with dexpanthenol they were significant after 10 days, and with *C. asiatica* and EHO-85 after 12 days. This suggests that each of these products can act on different phases of wound healing with different intensities, influencing the speed of wound closure in different ways. Under physiological conditions, hyaluronic acid (a natural polymer belonging to the group of glycosaminoglycans, actively involved in the formation of initial clots) is one of the main components of edema fluid, promoting the infiltration of neutrophils at an early stage and of leukocytes and monocytes at later stages of inflammation [21].

In addition, upon injury, transforming growth factor beta (TGF-β) in fibroblasts induces the synthesis and release of hyaluronic acid into the medium. This can bind to a cluster of differentiation 44 (CD44) in fibroblasts and activate extracellular signal-regulated kinase (ERK) and calcium/calmodulin (CaM)-dependent protein kinases, which induce expression of alpha smooth-muscle actin (α-SMA) and differentiation into myofibroblasts [39]. They, are primarily responsible for wound retraction [2]. Therefore, hyaluronic acid treatment can enhance myofibroblast formation and thus accelerate wound closure after six days of treatment, compared to other treatments.

On the other hand, dexpanthenol is characterized by the fact that it is rapidly absorbed by the skin and is transformed into pantothenic acid, which has important physiological functions in the epithelium [22]. Furthermore, dexpanthenol has important moisturizing properties and activates fibroblast proliferation and migration in wounds [40]. That has been related to its effects on accelerating wound closure [41].

In relation to the other two treatments, it is interesting to note that both have extracts of plant origin with high antioxidant power as active principles [14,42]. Both *Centella asiatica* and olive-tree leaf extracts have been described as having anti-inflammatory effects [42,43]. They may play a role in the early stages of healing, probably in conjunction with other important activities in this phase, such as cell proliferation and migration processes. Nevertheless, their overall effects are less potent than those produced with hyaluronic acid or dexpanthenol. This could explain the delay observed in wound closure for the former. However, more detailed studies on the mechanism of action, at the molecular and cellular levels, of each of the treatments would be necessary to obtain a conclusive explanation.

At the final study time, the percentage of wound closure was similar among all studied treatments. Nevertheless, histological analyses after seven and 14 days revealed some differences between them. Thus, after seven days, wounds treated with dexpanthenol had the best epithelialization, less cicatricial tissue depth, a lower number of inflammatory cells, and less vascularization. These results are in agreement with the known effects of dexpanthenol on healing [41]. In fact, dexpanthenol has been used in several studies as a reference to study the effects of different formulations or products on healing [37,44]. Dexpanthenol is a stable alcoholic analog of pantothenic acid, a member of the B-vitamin complex (vitamin B5), and a constituent of coenzyme A. Unlike pantothenic acid, dexpanthenol can be absorbed through the skin. Coenzyme A in the stratum corneum of the epidermis catalyzes the synthesis of fatty acids and sphingolipids. They are essential for maintaining the physiological functions of the skin. Due to its role in coenzyme A synthesis, pantothenic acid derived from dexpanthenol promotes epithelialization [22]. Furthermore, cream-applied dexpanthenol has anti-inflammatory effects [45]. Therefore, these data support our results on the favorable evolution at the histological level, of wounds treated with dexpanthenol, after seven days of healing.

With regard to the other treatments, after seven days, histological changes were only observed in samples derived from wounds treated with *Centella asiatica* and EHO-85, related to deep vascularization. These two treatments, as well as dexpanthenol, decreased the number of vessels with respect to the control wounds. The number of vessels in wounds increased at the beginning of healing, favoring the supply of nutrients and oxygen for tissue regeneration. However, with the maturation and remodeling of newly formed tissue, the number of vessels decreased. Indeed, it has been observed that there was a regression of vascularization in mouse wounds after seven days of treatment using micro-computed tomography (micro-CT) angiography techniques [46]. Therefore, our results suggest that vascular regression started earlier in wounds treated with EHO-85, dexpanthenol, or *Centella asiatica* than in untreated wounds, after seven days of healing. This would be an indicator that the regenerated tissue is at a more advanced stage of maturation with these treatments.

Wounds treated with EHO-85 also showed a higher degree of epithelialization than controls. This is in agreement with our previous results. Thus, the antioxidant activity of OELE protects human epidermal keratinocyte (HaCaT) cells from oxidative stress, increasing their viability in vitro after treatment with hydrogen peroxide [28]. Indeed, at early stages of healing, during inflammation, inflammatory cells produce high levels of reactive oxygen species (ROS). They protect against microbial infections. Subsequently, with the progression of the healing process, both inflammation and oxidative stress in the tissue decrease. That favors the proliferation and migration of endothelial cells, fibroblasts, and keratinocytes. Interestingly, while excess ROS negatively affects these cells, moderate and low levels of oxidative stress act as inducers of their regenerative activity [47]. In previous studies, we have found that EHO-85 treatment of wounds in rats decreased lipid peroxidation after 48 and 96 h of healing, in addition to increasing the ratio of reduced/oxidized glutathione (GSH/GSSG) after 96 h [14]. Therefore, a rapid decrease in oxidative stress in wounds treated with EHO-85 may favor an earlier onset of the proliferative phase of healing and promote re-epithelialization.

Histological analyses after 14 days showed that epithelialization was practically complete in all treatments. There were no significant differences in the depth of cicatricial tissue. However, wounds treated with EHO-85 showed the best results in parameters related to maturity and the formation of extracellular matrix. Thus, these wounds showed a lower number of vessels and inflammatory cells and higher levels of collagen in the extracellular matrix. At this time of healing, remodeling of the extracellular matrix predominates, while inflammation and the number of vessels continue to decrease [2,46].

The antioxidant effects of OELE may be involved in inflammation reduction after EHO-85 treatment. High levels of ROS cause tissue damage, intensifying the inflammatory response. In this sense, various antioxidant compounds, such as carotenoids and polyphenols, can regulate ROS levels. They may decrease inflammation through repression of transcription of genes encoding inflammatory factors as well as cytokines such as interleukin 1 (IL-1), IL-6, and tumor necrosis factor alpha (TNF-α) through the nuclear factor kappa-beta (NF-κβ) pathway [48]. Specifically, the OELE used as the active principle of EHO-85 has anti-inflammatory activity, as described by different studies. Thus, it has been demonstrated that OELE can regulate immune responses in humans by increasing the number of CD8+ and natural killer (NK) cells, inducing interferon-gamma (IFN-γ) production, and maintaining the balance between regulatory thymus (T) and T-helper 17 (Th17) cells [15].

Additionally, in vivo, OELE has been shown to prevent gastric mucosal damage in rats exposed to HCl/ethanol [49]. Also, they have the ability to induce polarization of M1 inflammatory macrophages into M2 [50]. The latter have less inflammatory activity, being involved in tissue-regeneration induction and favoring, among other actions, proliferation, migration, and collagen synthesis in fibroblasts [51]. Interestingly, most of the anti-inflammatory capacity of OELE is due to oleuropein [20]. This polyphenol is the most abundant in such extracts. Its anti-inflammatory activity has been demonstrated in different experimental models and pathologies [52]. In this context, all these data support the idea that the presence of OELE in EHO-85 may be the main cause of the decrease in inflammatory cells observed in wounds treated with this hydrogel.

On the other hand, fibroblasts are the main source of collagen synthesis in the dermis [2]. Thus, the increased accumulation of collagen in the extracellular matrix with EHO-85 treatment after 14 days of healing is likely due to the effect of such an amorphous hydrogel on fibroblasts and their activity. That is supported by previous results from our group, showing that OELE protects dermal fibroblasts in vitro against oxidative stress induced by hydrogen peroxide [28]. Also, other studies have shown the ability of OELE to act as a photoprotective, anti-inflammatory, and antioxidant agent in dermal fibroblasts when exposed to ultraviolet irradiation [53]. In addition, some studies indicate that oleuropein, which, as discussed above, is the most abundant polyphenol in OELE, has positive effects on fibroblasts. Thus, collagen-enriched nanovesicles containing oleuropein promote fibroblast proliferation and migration, which can accelerate skin wound healing [54]. Indeed, in incisional wounds in Bagg albino c (BALB/c) mice, intradermal injection of oleuropein accelerated skin healing after seven days. Histological analyses of wounds in that study showed that oleuropein-treated wounds had lower inflammatory cell infiltration, higher collagen content and better re-epithelialization [19]. These data are in agreement with our results, although the method of application was different. This suggests a key role for oleuropein in OELE as an inducer of healing.

## 4. Materials and Methods

### 4.1. Animals, Surgical Procedures and Treatments

All experimental procedures with animals were performed at the Experimental Animal Service (“Servicio de Animales de Experimentación”; SAEX) of the University of Cordoba, after approval on October 16, 2017, by the bioethics and biosafety committee of such a university and authorization by the Junta de Andalucía (File No. 16/10/2017/138). A total of 40 male Wistar rats, approximately weighing 350 g, were housed in individual cages in temperature-controlled rooms at 22 ± 3 °C and a relative humidity of 30–70%, under a 12/12 h light/dark photoperiod. They were fed a standard rodent diet and had access to water ad libitum.

The rats were divided into five experimental groups of eight animals each: (i) control; (ii) hyaluronic acid (Rym Cicatrizante, Ern. Madrid. Spain); (iii) *Centella asiatica* actives (Blastoactiva, Almirall. Barcelona. Spain); (iv) dexpanthenol (Bepanthol, Bayer. Barcelona. Spain); and (v) amorphous hydrogel EHO-85. The characteristics of the latter have been previously described. It consists of a hydrogel composed of geogard ultra, carbopol 980, disodium ethylenediaminetetraacetic acid (Na_2_-EDTA), trieathanolamine, glycerin, and fucocert, plus 0.1% OELE [28]. The rheological properties of EHO 85 have also been recently reported [55]. Additionally, the phenolic composition of OELE has been determined by liquid chromatography tandem mass spectrometry (LC-MS/MS) [28]. Results showed that it contained the following compounds: methyl (2S,3Z,4S)-4-{2-[2-(3,4-dihydroxyphenyl)ethoxy]-2-oxoethyl}-3-ethylidene-2-{[(2S,3R,4S,5S,6R)-3,4,5-trihydroxy-6-(hydroxymethyl)oxan-2-yl]oxy}-3,4-dihydro-2H-pyran-5-carboxylate (oleuropein), representing 78.07% of the phenolic content, followed by 2-(3,4-dihydroxyphenyl)ethyl 3-O-(6-deoxy-alpha-L-mannopyranosyl)-4-O-[(2E)-3-(3,4-dihydroxyphenyl)-2-propenoyl]-beta-D-glucopyranoside (verbascoside; 5.84%), 2-[(3Z)-3-ethylidene-5-(methoxycarbonyl)-2-{[3,4,5-trihydroxy-6-(hydroxymethyl)oxan-2-yl]oxy}-3,4-dihydro-2H-pyran-4-yl]acetic acid (oleoside-11-methyl ester; 5.41%), oleoside, dimethyl oleuropein, and 2-(3,4-dihydroxyphenyl)-5,7-dihydroxy-4H-1-benzopyran-4-one (luteolin)-7-glucoside, with proportions between 2 and 3%. Other compounds are polyphenols like 4-(2-hydroxyethyl)benzene-1,2-diol (hydroxytyrosol) glucoside, hydroxytyrosol, p-dihydroxyphenylacetic acid (p-DOPAC), 2-(3,4-dihydroxyphenyl)-5,7-dihydroxy-4-oxo-4H-chromen-3-yl 6-O-(6-deoxy-alpha-L-mannopyranosyl)-beta-D-glucopyranoside (rutin), (2R,3R)-2-(3,4-dihydroxyphenyl)-5,7-dihydroxy-4-oxo-3,4-dihydro-2H-chromen-3-yl 6-deoxy-alpha-L-mannopyranoside (astilbin), α-(2R,3R)-2-(3,4-dihydroxyphenyl)-3,5,7-trihydroxy-2,3-dihydro-4H-chromen-4-one (α-taxifolin), [4′,5,7-trihydroxyflavone, 5,7-dihydroxy-2-(4-hydroxyphenyl)-4H-1-benzopyran-4-one] (apigenin)-7-glucoside, p-hydroxyphenyl-ethyl alcohol (tyrosol) linked to 2-[(2S,3S,4S)-3-formyl-5-methoxycarbonyl-2-methyl-3,4-dihydro-2H-pyran-4-yl]acetic (elenolic) acid (p-HPEA-EA), 3,4-HPEA-EA (isomer of oleuropein aglycone), and 2-(3,4-dihydroxyphenyl)-5,7-dihydroxy-4H-1-benzopyran-4-one (luteolin), representing between 0.1 and 0.7% [28].

For wound generation, animals were anesthetized with isoflurane. Then, they were placed in a prone position to remove hair from the dorsal face with electric clippers. Subsequently, the area was cleaned with physiological saline solution, and the rats were placed in a lateral decubitus position to make two wounds on the dorsum with the help of a 15-mm disposable biopsy punch. An analgesic (bupaq 0.3 mg/mL) from Richter Pharma (Wels, Austria) was subcutaneously injected to alleviate possible post-surgical discomfort. Then, the different treatments were topically applied to each group of animals. The control animals were untreated rats. To avoid animal interactions on the wounds, once the treatments were applied, they were covered with a silicon foam dressing (mepilex lite), from Molnlycke (Gothenburg, Sweden). It was held in place by a latex-free tearable cohesive dressing (cpk sport) from Farmaban (Fruitos de Bages, Spain) to avoid animal-induced detachment. Every two days, the dressing was removed, the wound was cleaned with physiological saline solution, and the corresponding treatment was applied. After seven and 14 days after the onset of healing and treatments, four animals from each group were sacrificed by exsanguination and overdose of anesthesia (sodium pentobarbital or dolethal) from Vetoquinol (Paris, France). Both wounds were excised for fixation, paraffin inclusion, and subsequent histological analyses.

### 4.2. Measurements of Wound-Size Reductions

Postoperative photographs of wounds were taken with a digital camera, using a millimeter ruler as a reference, at time 0 and every two days. Changes in wound areas were analyzed using ImageJ software version 1.53f51 from the National Institutes of Health (NIH; Bethesda, MD, USA). The percentage reduction in wound size was calculated using the formula: [(A_0_ − A_t_)/A_0_] × 100, where A_0_ is the initial wound area and A_t_ is the wound area at each time point.

### 4.3. Histological Analyses

The skin samples corresponding to the wounds after seven and 14 days were dehydrated, embedded in paraffin, and cut into ~5 μm-thick sections. These were stained with hematoxylin and eosin. In short, sections were stained with hematoxylin, then with eosin, dehydrated in ethanol with a concentration gradient, and, finally, with xylol. Samples were sealed with Eukitt mounting medium for subsequent analyses under an optical microscope. All chemicals were from Illinois Tool Works (ITW) Reagents (PanReac-AppliChem, Barcelona, Spain]. Histological analyses of H & E-stained sections consisted of quantification of percentages of epithelialization, epidermal thickness, depth of cicatricial tissue, and number of infiltrating inflammatory cells, as well as vascularization (number of vessels), both subepidermally and in-depth.

In order to evaluate the state of the extracellular matrix and its collagen content in the later stages of healing, sections of healing tissues after 14 days of treatments were also stained with Masson’s trichrome from ScyTek Laboratories (Logan, UT, USA). In short, sections were stained with Weigert’s ferric hematoxylin for three min, rinsed, and stained with Biebrich Scarlet/Acid Fuchsin for 5 min. Then, samples were placed in phosphomolybdic/phosphotungstic acid for 10 min, stained with aniline blue for five min, and washed with 1% acetic acid for three min. They were dehydrated in ethanol with a concentration gradient and finally washed in xylol. Samples were sealed with Eukitt for subsequent analyses. Images obtained by optical microscopy were analyzed with ImageJ software for the following parameters: area, intensity, and density of collagen staining.

### 4.4. Statistical Analyses

Variables were expressed as means and standard deviations (SD). Comparisons between groups with respect to wound-healing activity were analyzed with an analysis of variance (ANOVA) and Fisher’s least significant difference (LSD) multiple comparison test. Differences were considered significant for *p* < 0.05. Statistical analyses were performed with Prism 8.0 software from GraphPad Software (San Diego, CA, USA).

## 5. Conclusions

This work had a double objective. On the one hand, to test the efficacy of EHO-85 for the treatment of cutaneous wounds in comparison to other products normally used in human clinical practice for this purpose around the world, and, on the other hand, to deepen the mechanisms of action of this hydrogel through histological evaluations over time of the treated wounds. Regarding the first objective, the study has shown that treatment with EHO-85 is equal to or superior to the other treatments. Therefore, this indicates that this hydrogel containing OELE (a biocompatible, accessible, and low-cost natural extract from olive tree leaves) is a suitable option for the treatment of skin wounds. In relation to the histological analyses performed, we can conclude that EHO-85 has a more potent anti-inflammatory effect, which helps prevent wound chronification, and a greater ability to promote extracellular matrix synthesis and remodeling of regenerated tissue. This supports and helps explain the positive effect that this hydrogel has on skin ulcers in patients with different etiologies [29].

Thus, EHO-85 is, to the best of our knowledge, one of the few hydrogels containing natural compounds as an active principle that has been evaluated in both preclinical and clinical trials on wound healing, with positive results in both cases. Therefore, based on the results of the present study, which show that the effect of EHO-85 is not inferior, and even superior in some aspects, to other products used in the human clinic, we can conclude that it is a product with a high potential for translation to clinical use and could be proposed as a suitable option for the treatment of skin wounds.

## Data Availability

The data presented in this study are available on request from the corresponding author.
